# Sexually Transmitted *Treponema pallidum* Subspecies *endemicum* Infection With Atypical Skin Manifestations Outside of Tropical Regions

**DOI:** 10.1093/ofid/ofaf019

**Published:** 2025-01-10

**Authors:** Eisuke Adachi, Wakana Sato, Hirona Ichimura, Yasutoshi Kido, Hiroshi Yotsuyanagi

**Affiliations:** Department of Infectious Diseases and Applied Immunology, IMSUT Hospital of the Institute of Medical Science, University of Tokyo, Tokyo, Japan; Department of Infectious Diseases and Applied Immunology, IMSUT Hospital of the Institute of Medical Science, University of Tokyo, Tokyo, Japan; Department of Virology and Parasitology, Graduate School of Medicine, Osaka Metropolitan University, Osaka, Japan; Department of Virology and Parasitology, Graduate School of Medicine, Osaka Metropolitan University, Osaka, Japan; Department of Infectious Diseases and Applied Immunology, IMSUT Hospital of the Institute of Medical Science, University of Tokyo, Tokyo, Japan

To the  Editor—Endemic treponematoses, such as yaws, bejel, and pinta, are classified as neglected tropical diseases and are often referred to as non–sexually transmitted syphilis. Bejel remains prevalent in dry, hot regions such as the Sahel in West Africa, with its primary route of transmission being direct skin-to-skin contact in children [1]. Although sexual transmission is possible, it has not been extensively studied because bejel primarily affects children. Due to the low genetic differences between *Treponema pallidum* subspecies *pallidum* (TPA) and other non–sexually transmitted syphilis-causing strains, identifying them without high-resolution phylogenetic analysis can be challenging. The difficulty in culturing *T pallidum* in the laboratory has posed significant challenges for molecular epidemiological analysis. Little is known about its clinical manifestations and prevalence outside tropical regions.

A 67-year-old Japanese man who has sex with men (MSM) with human immunodeficiency virus (HIV) on antiretroviral therapy presented with a systemic skin rash. His CD4 cell count was 198 cells/μL and HIV RNA was undetectable. He exhibited diffuse papular erythema on his trunk and extremities (Figure 1). Laboratory tests revealed a rapid plasma reagin (RPR) titer of 1432 RU (RPR units, measured using the LASAY Auto RPR system from SHIMA Laboratories Co) and a *T pallidum* latex agglutination of 691 750 U/mL, leading to a diagnosis of syphilis based on initial clinical findings. He received 1500 mg of amoxicillin daily for 30 days, leading to an improvement in his rash, and his RPR levels decreased. However, nested polymerase chain reaction analysis using whole blood samples detected 3 loci (*TP0136, TP0548*, and *TP0705*), and multilocus sequence typing failed to match any sequence type of TPA [2, 3]. Using the Basic Local Alignment Search Tool (BLAST) analysis of the *TP0136* and *TP0548* loci, the strain was identified as *T pallidum* subspecies *endemicum* (TEN), the causative agent of bejel [3]. Although this infection could be treated similarly to TPA with amoxicillin, its clinical presentation differed. Instead of roseola, the patient showed characteristic skin findings specific to TEN.

**Figure 1.**
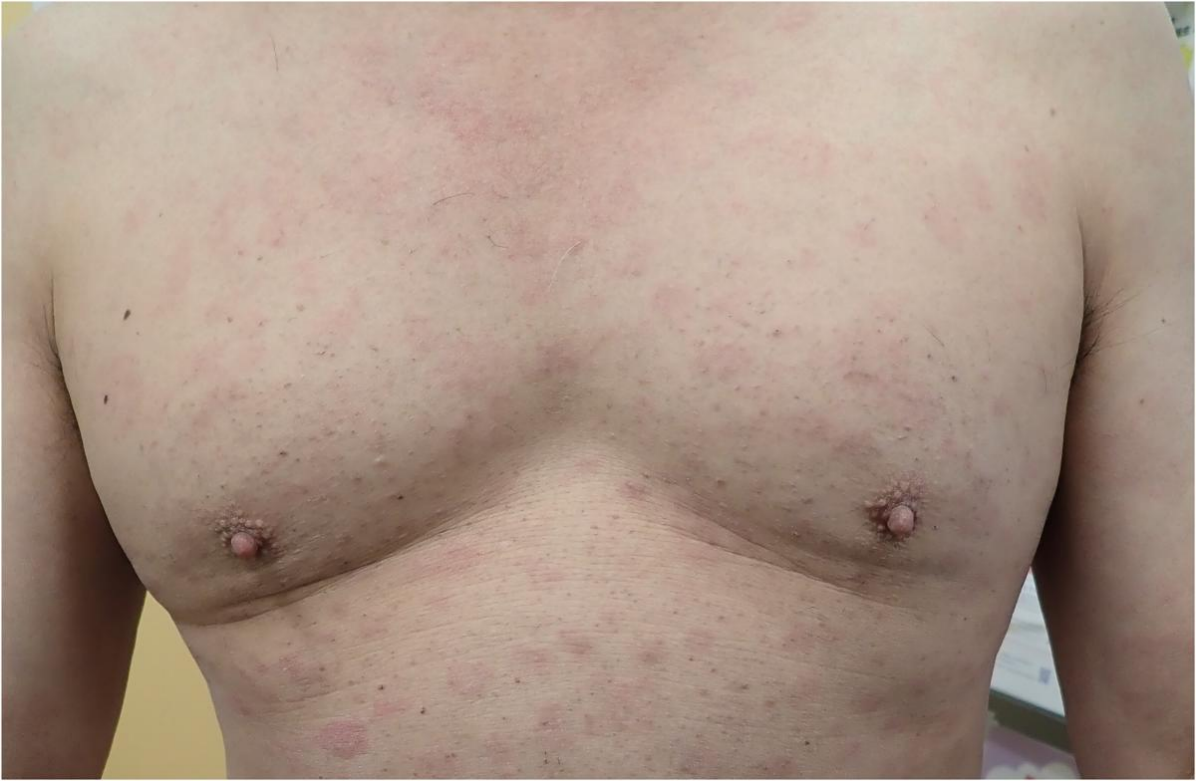
Diffuse papular erythema in an Asian patient with bejel living in a nontropical area.

Previously, TEN had been detected in stored blood samples from patients living in Japan outside of tropical regions [4]. According to a report, the *T pallidum* strains prevalent among MSM with HIV in Japan, treated as syphilis in routine practice, include not only the SS14-like strain, which is most common in Europe [5], but also the Nichols-like strain, with an additional 12% being TEN [3]. However, clinical information, particularly regarding skin manifestations, has been limited. Given that the patient had no history of travel to endemic areas, it is strongly suspected that the infection was acquired through sexual transmission within Japan. This suggests that individuals diagnosed with syphilis could potentially include those with bejel, indicating that TEN might be sexually transmitted among Japanese MSM. While this case demonstrates that bejel, like syphilis, causes elevated RPR titers that decrease with treatment, identifying any clinical findings unique to bejel as a sexually transmitted disease that are not typically observed in syphilis would be valuable. Such distinctions could provide important insights into the presentation and management of bejel in nonendemic areas.

## Notes

***Author contributions.*** E. A. and H. Y. were responsible for the clinical management of the patient. H. I., W. S., and Y. K. performed the genome analysis and identified TPN. E. A. drafted the manuscript. All authors approved the final manuscript.

***Ethics approval.*** Ethics approval was granted by the ethics board of the Institute of Medical Science, University of Tokyo (2020-79-0324).

***Patient consent.*** The patient gave consent for publication.
